# Latitudinal distribution of microbial communities in anaerobic biological stabilization ponds: effect of the mean annual temperature

**DOI:** 10.1111/1751-7915.12407

**Published:** 2016-08-26

**Authors:** Mengdong Yuan, Jing Zhu, Cheng Wang, Mengxiong Wu, Faqian Sun, Xingguo Han, Yangyang He, Weixiang Wu

**Affiliations:** ^1^ Institute of Environmental Science and TechnologyZhejiang UniversityHangzhou310058China

## Abstract

Considering wide utilization and high methane fluxes from anaerobic biological stabilization ponds (ABSPs), understanding the methanogenesis in ABSPs is of fundamental importance. Here we investigated the variation and impact factors of methanogenesis in seven ABSPs that spanned from the north to the south of China. Results showed that methanogen abundance (7.7 × 10^9^–8.7 × 10^10^ copies g^−1^ dry sediment) and methanogenic activities (2.2–21.2 μmol CH_4_ g^−1^ dry sediment h^−1^) were considerable for all sediments. Statistical analysis demonstrated that compared with other factors (ammonium, pH, COD and TOC), mean annual temperature (MAT) showed the lowest *P* value and thus was the most important influencing factor for the methanogenic process. Besides, with the increasing MAT, methanogenic activity was enhanced mainly due to the shift of the dominant methanogenic pathway from acetoclastic (49.8–70.7%) in low MAT areas to hydrogenotrophic (42.0–54.6%) in high MAT areas. This shift of methanogenic pathway was also paralleled with changes in composition of bacterial communities. These results suggested that future global warming may reshape the composition of methanogen communities and lead to an increasing methane emission from ABSPs. Therefore, further research is urgently needed to globally estimate methane emissions from ABSPs and re‐examine the role of ABSPs in wastewater treatment.

## Introduction

Anaerobic biological stabilization ponds (ABSPs) are widely used for wastewater treatment around the world due to their simple operation, effective cost and low maintenance requirements. Particularly, in regions where land is readily available (Kivaisi, 2001), such as non‐metropolitan and regional USA, Australia, central Europe and China, ABSPs are the preferred wastewater treatment process. For swine farms located far from the city with large areas of available land, ABSPs have been recommended as the most effective wastewater treatment process for the removal of chemical oxygen demand (COD) (Liu *et al*., 2014). However, a considerable amount of greenhouse gas could emit from these uncovered ponds. It has been reported that methane flux of ABSPs in North America (Adler, 1994), New Zealand (Park and Craggs, 2006), French Mediterranean coast (Picot *et al*., 2003) and Mexico (Hernandez‐Paniagua *et al*., 2014) ranged from 7.7 to 64 g m^−2^ d^−1^, which were much higher than that of paddy soils (0.092–1.1 g m^−2^ d^−1^) (Seiler *et al*., 1983) and wetlands (0.048–0.71 g m^−2^ d^−1^) (Baker‐Blocker *et al*., 1977). Moreover, a long‐term input of high concentration of COD and an anaerobic condition of ABSPs sediments could be expected to promote the degradation of organic matters (Toprak, 1995), which would lead to an increase of methane emissions and thereby accelerated global warming. Despite the environmental and climate importance of this process, the composition of functional microbial community structure, especially methanogens in ABSPs is still unclear. What is more, little is known about the influence of biotic (syntrophic‐related bacteria) (Fotidis *et al*., 2014) and abiotic drivers on the methanogenesis in ABSPs.

Temperature, in particular, has been implicated as a major factor in methane emission and methanogenesis. Based on a database of 1553 measurements of methane emission and temperature across wetlands, rice paddies and aquatic ecosystems, meta‐analysis revealed that both methane emission and ratio of methane to CO_2_ emission markedly increased with increasing seasonal temperature (Yvon‐Durocher *et al*., 2014). In addition, this temperature dependence was consistent with the catabolism of methanogens and composition of methanogen communities. First, the precursors of methanogenesis were accumulated in the peatland during colder periods of the year and then metabolized via acetoclastic methanogenesis during warmer seasons (Shannon and White, 1996). The turnover rate of acetate increased from 3 to 26 nmol l^−1^ h^−1^ with temperature increasing from 4 to 25°C (Kotsyurbenko *et al*., 2004). Second, the composition of methanogenic community was affected by temperature variation: relative abundance of Methanocella increased at 15°C while that of Methanoregula and Methanosaeta increased at 5°C (Schmidt *et al*., 2015). However, previous studies mainly focused on paddy soil, wetland and uplands. Systematic research into the variation of methane emission and methanogenesis under different temperature conditions in ABSPs does not appear to have been carried out. To date, only three studies mentioned the temperature conditions in their research about methane emission from ABSPs (Picot *et al*., 2003). About 2.3‐ to 5.2‐fold higher methane flux was observed in the ABSPs with an average temperature of 37°C than 15°C (Park and Craggs, 2006). It could be postulated that elevated temperature would also increase the methane emission and impact on the methanogenesis in ABSPs. However, further research is needed to confirm this influence and explore the underlying mechanisms – variability of methanogenic community diversity and structure in response to temperature change.

**Figure 1:**
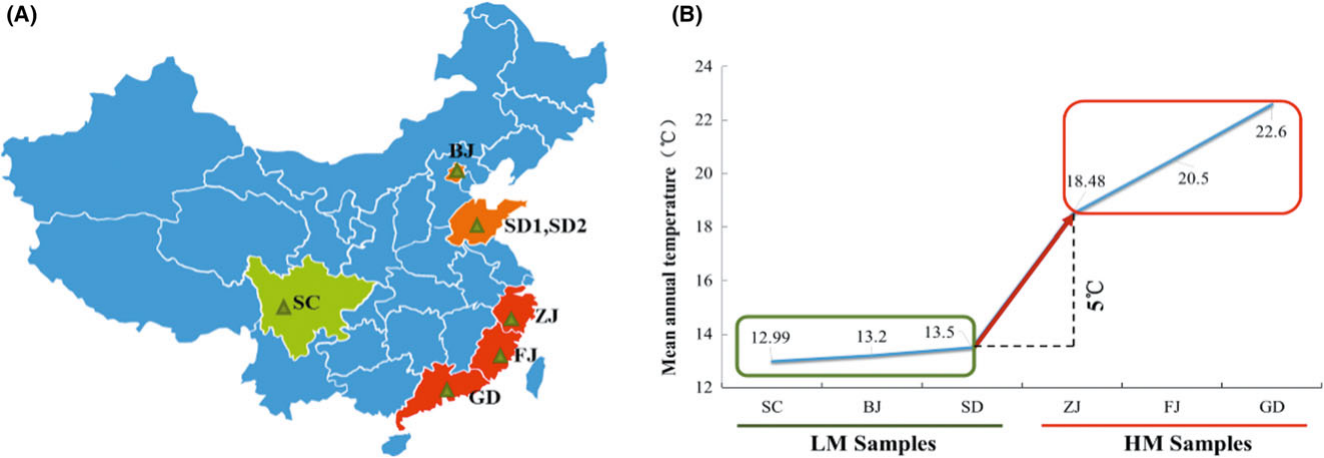
Sampling sites. (A) The geographical location (this figure was generated using Microsoft Powerpoint 2013 and Adobe Photoshop 7.0). (B) MAT (12.9–20.6°C).

China spans a latitude gradient of 50° and covers a wide range of climate zones with a large mean annual temperature (MAT) gradient from north to south. This MAT gradient provides an ideal environment to investigate the distribution of microbial community structure in ABSPs under different MAT conditions. In this study, sediment and water samples were collected from ABSPs for piggery wastewater treatment across six provinces with different latitudes in China in 2014 (Fig. 1A). From north to south, sampling sites were located in Beijing (BJ, N40°12′0.14″ E116°33′45.18″), Shandong (SD1, N36°04′25.1″ E116°43′08.2″ and SD2, N36°03′24.7″ E116°38′26.1″), Zhejiang (ZJ, N30°24′40.22″ E119°54′2.90″), Sichuan (SC, N30°14′31.54″ E103°34′38.14″), Fujian (FJ, N24°24′44″ E118°04′07″) and Guangdong (GD, N21°52′4.94″ E111°57′55.76″), with significantly different MATs (Fig. 1B) (China Meteorological Administration, 2004‐2013). Based on the differences in MAT (more than 5°C), these sites were divided into a low‐MAT (LM) area (SC, BJ, SD1 and SD2) and a high‐MAT (HM) area (ZJ, FJ and GD). In addition, the relationship between environmental variables (i.e. pH, ammonium, nitrite, nitrate, COD, TOC, water content and MAT) and microbial community distribution was investigated in order to elucidate whether MAT was an influential environmental factor. These results are important to our understanding of methanogenesis in ABSPs along a MAT latitude gradient. Moreover, even though some research concerning the influence of temperature on methane emission has been reported (Toprak, 1995), the research on the underlying mechanism, especially microbial community composition related to the methanogenesis is still lacking. Further study about microbial community related to methanogenesis will help us better estimate the influences of climate change on the methane emission from ABSPs in the future.

## Results and discussion

### Methanogenic activity in ABSPs

Serum bottle incubation experiments showed that the mean methanogenic activity of all sediment samples was 7.33 μmol CH_4_ g^−1 ^dry sediment h^−1^, ranging from 2.2 to 21.25 μmol CH_4_ g^−1 ^dry sediment h^−1^. Compared with the mean methane oxidation activity (0.7 μmol CH_4_ g^−1 ^dry sediment h^−1^), methanogenic activity was 10.1‐fold higher (Table 1). Compared with paddy soil (< 0.02 μmol CH_4_ g^−1 ^dry sediment h^−1^) (Dong *et al*., 2013), ABSPs sediments in our study also showed a much higher methanogenic activity.

**Table 1:** Physicochemical characteristics of the sediment samples

Sediment sample^a^	Ammonium (g kg^−1^)	Nitrite (mg kg^−1^)	Nitrate (g kg^−1^)	TOC (g kg^−1^)	pH	Water content	Methanogenic activity^b^	Methane oxidation activity^b^	MAT (2004–2013)
LM									
SC	3.38 ± 0.24	0.41 ± 0.06	0.08 ± 0.01	154.17 ± 9.44	8.30 ± 0.11	0.86 ± 0.01	2.83 ± 0.12	0.46 ± 0.05	12.99 ± 0.67
BJ	8.77 ± 0.02	0.22 ± 0.01	0.17 ± 0.04	202.45 ± 0.28	8.28 ± 0.11	0.88 ± 0.01	2.20 ± 0.07	0.49 ± 0.02	13.20 ± 0.57
SD1	3.58 ± 0.09	0.55 ± 0.02	0.09 ± 0.01	170.65 ± 9.19	8.04 ± 0.09	0.87 ± 0.03	2.91 ± 0.09	0.44 ± 0.03	13.50 ± 0.39
SD2	2.42 ± 0.04	0.28 ± 0.01	0.05 ± 0.00	173.22 ± 3.85	7.95 ± 0.06	0.85 ± 0.03	2.82 ± 0.19	0.62 ± 0.02	13.50 ± 0.39
HM									
ZJ	10.44 ± 2.06	1.84 ± 0.05	0.06 ± 0.00	186.70 ± 1.41	7.79 ± 0.07	0.91 ± 0.02	21.25 ± 3.97	0.77 ± 0.23	18.48 ± 0.59
FJ	7.36 ± 0.13	3.80 ± 0.06	0.05 ± 0.01	108.70 ± 6.78	8.34 ± 0.11	0.92 ± 0.01	8.92 ± 0.06	1.17 ± 0.07	20.50 ± 0.82
GD	12.65 ± 0.21	3.62 ± 0.64	0.11 ± 0.00	254.32 ± 5.16	8.46 ± 0.11	0.92 ± 0.02	10.38 ± 2.74	1.11 ± 0.16	22.60 ± 0.71

a Means ± standard deviation.

b The measurement unit is μmol CH_4_ g^−1^ dry sediment h^−1^.

**Figure 2:**
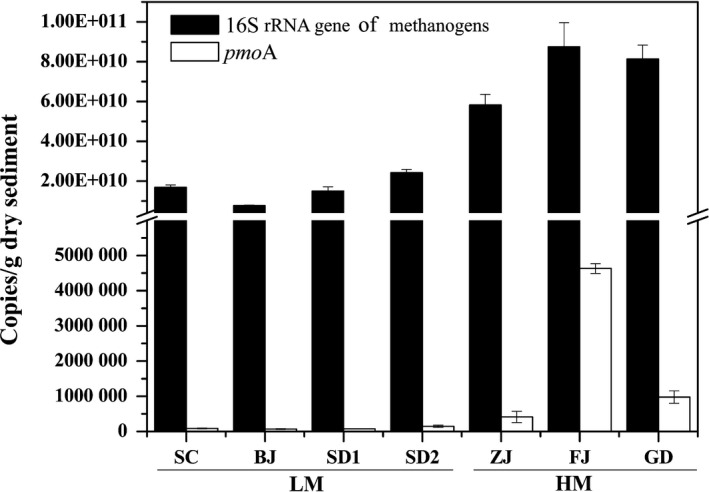
Abundance of 16S rRNA gene of methanogens and *pmoA* gene (copies g^−1^ dry sediment) in the sediment samples. Error bars indicate standard deviation of the mean of triplicate qPCR reactions.

The high methanogenic activities in ABSPs sediments can be attributed to the following reasons. First, the high carbon and nitrogen concentration of water and sediments could provide enough substrate for methane production (Zitomer and Shrout, 2000). Physicochemical analyses revealed that almost all of the ABSPs had a high content of COD (382.3–973.3 mg l^−1^) (Table S1) and high ammonium (166.7–715.1 mg l^−1^) in water samples and high total organic carbon (108.7–254.3 g kg^−1^) and ammonium (2.42–12.65 g kg^−1^) (Table 1) in sediments. Additionally, the low dissolved oxygen concentrations (0.15–0.23 mg l^−1^) (Table S1) of the ABSPs water suggested that an anaerobic environment existed in the ABSPs, which provides a suitable habitat for methanogens (Großkopf *et al*., 1998). As expected, Illumina Miseq sequencing of archaea targeting V5‐V6 region revealed that methanogens accounted for more than 82.7% of the total archaea. Meanwhile, a small fraction of methane‐oxidizing archaea (< 0.07%) and bacteria (NA) was observed. qPCR results (Fig. 2, Table S2 & Fig. S2) showed that the abundances of 16S rRNA gene of methanogens in sediments and water were 1.89 × 10^4^–2.10 × 10^5^‐fold and 2.28–10.55‐fold higher than *pmoA* gene, respectively, which further demonstrated that the methanogenic process was predominant. In addition, the abundances of 16S rRNA gene of methanogens in the pond sediments were higher than observed in UASB sludge granules when treating swine wastewater (1.9 × 10^7^–5.7 × 10^7^ copies ml^−1^) (Song *et al*., 2010), anaerobic batch digesters (2.8 × 10^7^–4.5 × 10^8^ copies ml^−1^) (Lee *et al*., 2009) and fixed‐bed anaerobic digester (2.8 × 10^7^–7.6 × 10^9^ copies ml^−1^) (Sawayama *et al*., 2006). Totally, the high methanogenic activity and abundance of methanogens in our study suggested that considerable amount of methane would be emitted from the ABSPs for swine wastewater treatment. In addition, the activities in our research were not measured in situ but in the laboratory. Therefore, the measured activities might be different from the real methane emissions from ABSPs, which is recommended to be measured in the future.

### Microbial community diversity

A total of 172 993 and 205 918 sequences were obtained using the archaeal V5‐V6 primers and bacterial V4 primers respectively. The approach of an asymptote in the rarefaction curve indicated that the archaeal and bacterial community were well captured at the sequencing depth (Fig. S1). Besides, the non‐parametric statistics analyses (Table 2) showed that the observed sequences covered 46–52% and 62–79% of the total archaeal and bacterial sequences.

**Table 2:** Richness and diversity of archaea and bacteria in the sediment samples collected from ponds located from northern to southern parts of China (estimated by 97% OTU clusters)

Sediment sample	Archaea							Bacteria						
	Richness estimators				Diversity estimators			Richness estimators				Diversity estimators		
	S_obs_ a	Chao	ACE	Chao/ACE	Simpson	Shannon	% Inv. Comp.^b^	S_obs_ a	Chao	ACE	Chao/ACE	Simpson	Shannon	% Inv. Comp.^b^
LM														
SC	185	401.0	433	0.92	0.44	1.38	46	835	1322	1353	0.98	0.06	3.85	63
BJ	263	554.6	583	0.95	0.46	1.67	47	628	1018	963	1.06	0.13	3.17	62
SD1	266	542.3	561	0.97	0.30	1.92	49	1109	1549	1564	0.99	0.04	4.43	72
SD2	275	530.0	545	0.97	0.29	2.01	52	1055	1405	1446	0.97	0.04	4.30	75
HM														
ZJ	287	621.4	836	0.74	0.16	2.59	46	1457	1927	1931	1.00	0.02	5.21	76
FJ	287	622.6	739	0.84	0.14	2.84	46	1665	2104	2144	0.98	0.01	5.38	79
GD	267	582.4	700	0.83	0.19	2.29	46	1041	1427	1427	1.00	0.05	4.41	73

a Observed richness.

b Total observed richness/Chao estimate ×100.

**Figure 3:**
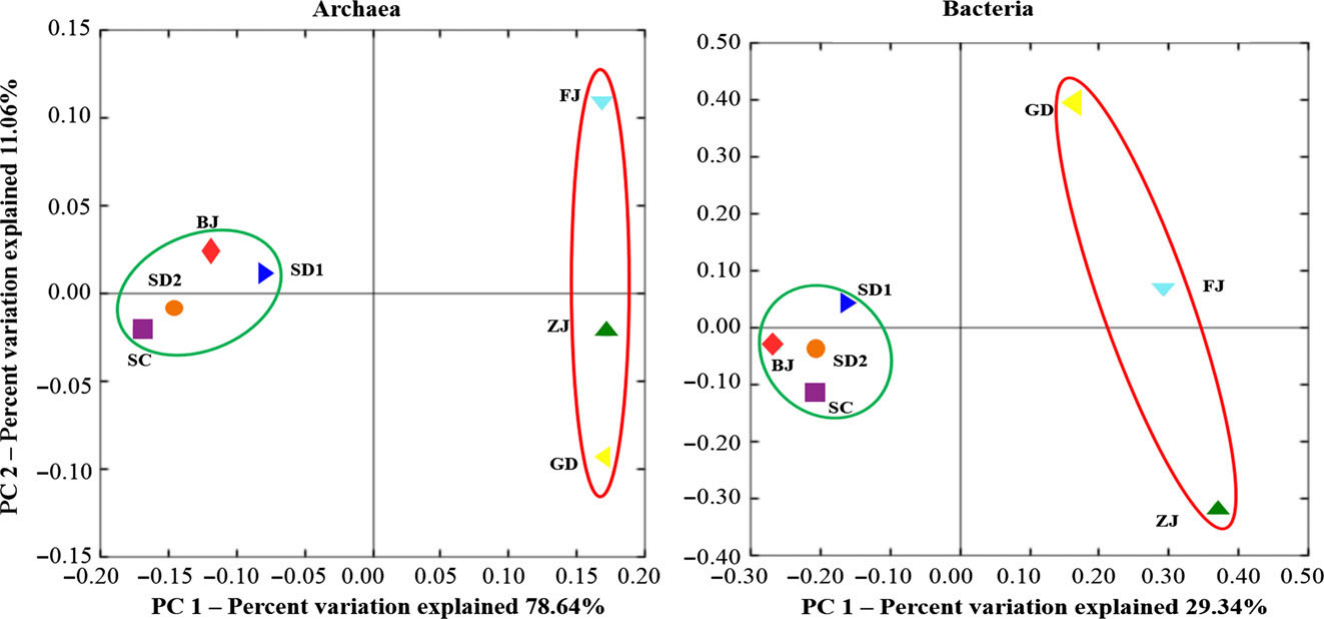
Principal coordinate analyses results of archaeal and bacterial communities in the sediment samples.

The microbial richness and diversity of the sediment samples from different ponds were compared using the Shannon, Simpson, S_obs_, Chao and ACE index (Table 2). For archaea and bacteria, the highest richness and diversity were both found in sediments collected from FJ and ZJ. The microbial richness and diversity increased with the increasing MAT. Furthermore, based on the explanation of 89% and 48% of total variation, the distribution of principal coordinate analyses (PCoA) plots (Fig. 3) revealed that the distributions of archaeal and bacterial communities in the sediment samples area were similar and could be clustered into LM and HM areas respectively.

### Bacterial community structure

**Figure 4:**
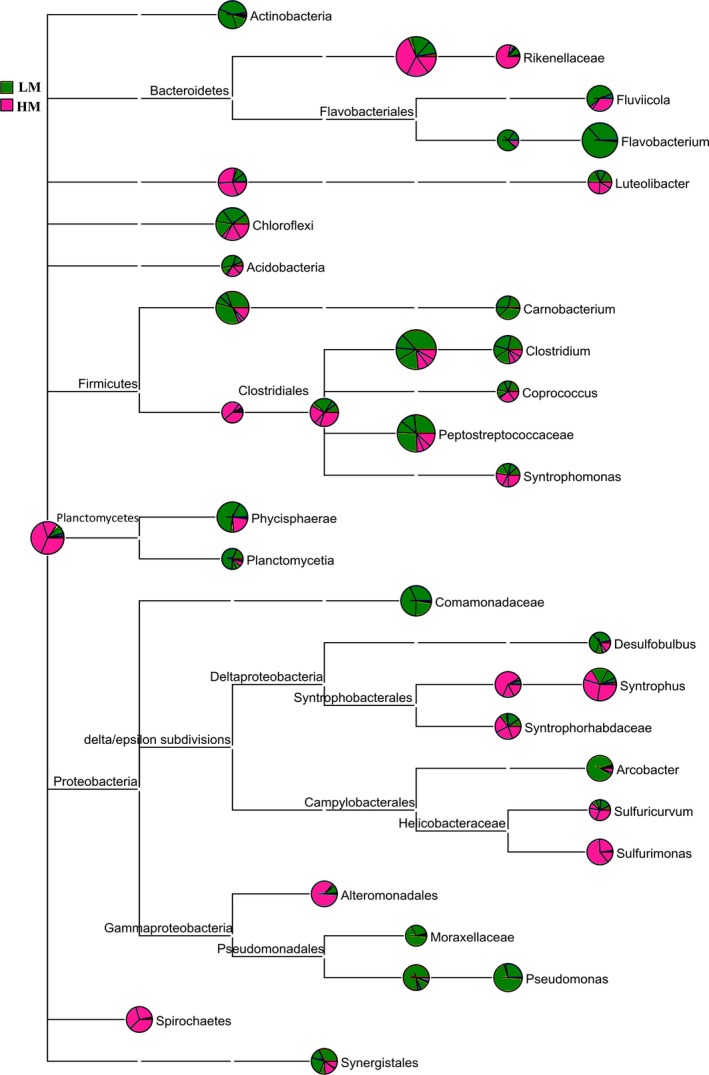
Comparison of bacterial community among sediment samples from the different sampling sites. The area of the circle represents the percentage of the species. Different colours show the sediment samples collected from different mean annual temperature areas.

A total of 20 major bacterial phyla (> 0.2%) were identified in all sediment samples, including *Firmicutes*,* Bacteroidetes*,* Proteobacteria*,* Chloroflexi*,* Planctomycetes*,* Verrucomicrobia*,* Actinobacteria*,* Spirochaetes*,* OP8*,* Synergistetes*,* OD1*,* Caldithrix*,* OP9*,* Hyd24‐12*,* NKB19*,* H‐178*,* Tenericutes*,* Lentisphaerae*,* Acidobacteria* and *Armatimonadetes*. The three predominant phyla in all sediment samples were assigned to *Firmicutes* (21.20–80.45%), *Proteobacteria* (5.43–18.46%) and *Bacteroidetes* (3.34–40.92%) (Fig. 4 & Table S3). The percentage of *Firmicutes* (mainly the families of Clostridiaceae and Peptostreptococcaceae) decreased while *Proteobacteria* (mainly the class of δ‐*Proteobacteria* in the sediment samples from HM area and mainly the classes of β‐*Proteobacteria*, γ‐*Proteobacteria* and ε‐*Proteobacteria* in the sediment samples from LM area) and *Bacteroidetes* (mainly the classes of *Bacteroidia*,* Flavobacteriia* and *Sphingobacterii*) increased with increasing MAT. In contrast, *Actinobacteria* and *NKB19* only existed in the sediment samples from LM area, while *OD1*,* Spirochaetes* and *Caldithrix* were only found in the sediment samples from HM area. *Synergistetes* showed a higher percentage in the sediment samples from LM area, whereas *Verrucomicrobia* was assigned a larger percentage in the sediment samples from HM area.

### Archaeal community structure

In accordance with the results of PCoA, the sediment samples shared similar archaeal community structure for HM area and LM area. Four different archaeal phyla including *Euryarchaeota*,* Parvarchaeota*,* Crenarchaeota* and a newly discovered *Thaumarchaeota* were detected in all sediment samples. *Euryarchaeota* (> 55%) was the most abundant phylum of archaea among all those sediment samples and this phylum showed an especially high abundance in the sediment samples from LM area (> 85.5%). *Parvarchaeota* showed a higher percentage in the sediment samples from HM area (> 14.34%) than LM area, while *Crenarchaeota* (4.5%) and *Thaumarchaeota* (0.04%) were evenly distributed in all sediment samples.

**Figure 5:**
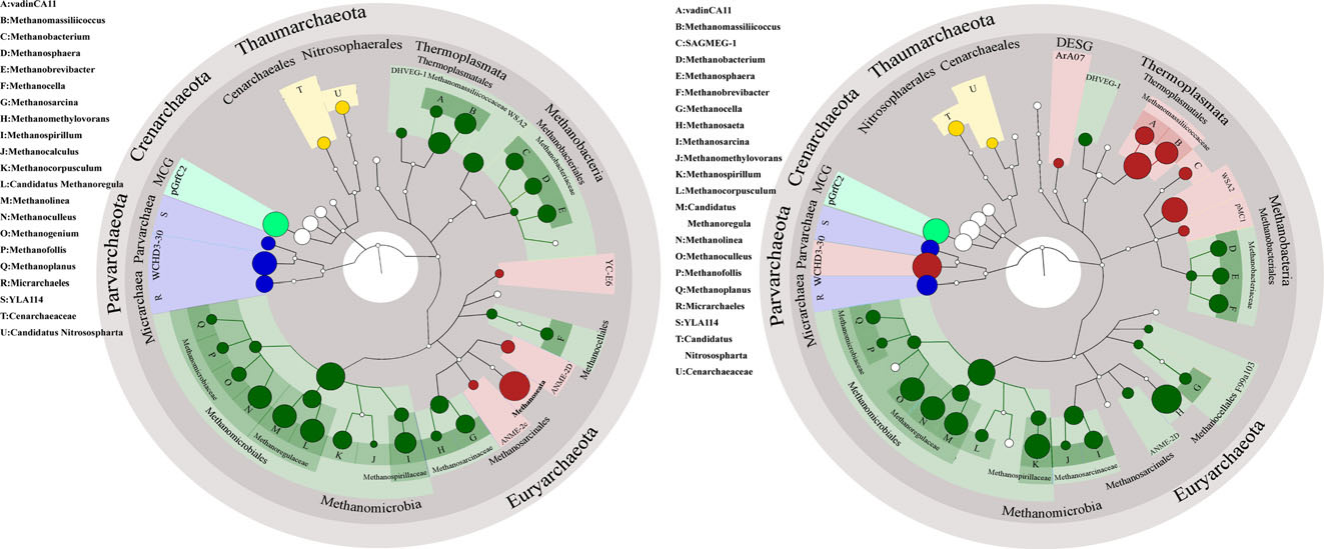
Comparison of archaeal community structure in the sediment samples collected from LM (left) and HM (right) area. For each figure, the archaeal composition at phylum, class, order, family and genus levels are shown from outside to inside of the circle. Each colour represents one phylum except the red one, which represents the typical species or obviously more abundant species than another.

More differences of the four achaeal phyla distribution were observed among sediment samples from different MAT area at deeper levels (Fig. 5 & Table S4). At the class level, all the observed archaea were classified into 12 classes. Methanomicrobia was the predominant class in all sediment samples, which showed a decreasing tendency with increasing MAT (> 84.5% and > 44.7% of archaea in the sediment samples from LM and HM areas respectively). In the sediment samples from HM area, *Parvarchaea* (> 14.2%), *Thermoplasmata* (> 7.9%) and *Methanobacteria* (> 1.8%) were widely observed while they showed much lower percentages in the sediment samples from LM area (< 5.74%, 1.55% and 0.69% respectively) than HM area. MCG was present in all sediment samples, which accounted for more than 2% of all the archaeal members. Many other little fraction classes (< 0.34%), including *Micrarchaea*,* DSEG*,* Nitrososphaerales* and *Thermoprotei*, were mostly found in the sediment samples from HM area. *Aigarchaeota*,* Halobacteria* and *Methanococci* were identified only in the sediment samples from HM area. At the order level, a total of 17 archaeal orders were identified in all sediment samples. On average, seven dominant orders (> 0.2%) belonged to *Methanosarcinales* (28–70%), *Methanomicrobiales* (13.5–34.5%), WCHD3‐30 (1.14–27.1%), *Thermoplasmatales* (0.68–11.4%), *pGrfC2* (2.16–6.65%), *Methanobacteriales* (0.37–5.34%) and *Micrarchaeles* (0–0.34%). The percentages of *Methanosarcinales*,* Methanomicrobiales* and *pGrfC2* decreased with the increasing MAT. The opposite tendency was observed in the orders of *WCHD3‐30, Thermoplasmatales* and *Methanobacteriales*. The orders of ArA07 and F99a103 only existed in the sediment samples from HM area while YC_E6 only existed in LM area. *Nitrosophaeraceae* (0.01–0.08%), unclassified *Thermoprotei* (0–0.07%), *Methanocellales* (0–0.04%) and *Cenarchaeales* (0.01–0.03%) were evenly distributed in all sediment samples. At the family level, 16 identified families and 10 unclassified families were present in all sediment samples. *Methanosaetaceae* (49.5–70.46%) and unclassified *Methanomicrobiales* (12.15–28.32%) were the predominant families and showed a higher percentage in the sediment samples from LM area than HM area, while unclassified WCHD3‐30 (13.99–27.14%) and *Methanomassiliicoccaceae* (7.84–11.39%) showed a higher percentage in the sediment samples from HM area. ANME‐2D and ANME‐2c were also detected in all sediment samples at this level.

### Controls on methanogenesis in ABSPs sediments

All the results confirmed our hypothesis that MAT played the most important factor in impacting on methanogenesis in ABSPs sediments. First, serum bottle incubation experiments and qPCR analysis suggested that the methane emission might be increased with an elevated temperature. A 5‐fold higher methanogenic activity was observed in the sediment samples from HM area compared with those from LM area (Table 1). Nevertheless, only 2.01‐fold higher methane oxidation activity was observed in the sediment samples from HM area than those from LM area. From the perspective of functional microbes abundance, the abundance of 16S rRNA gene of methanogens in the sediment from HM area were 4.74‐fold larger than LM area, while only 1.88‐fold larger was observed for the abundance of *pmoA* gene (Fig. 2). In the water, the abundance of 16S rRNA gene of methanogens from HM area were 21.11‐fold larger than LM area, while only 13.41‐fold larger was observed for the abundance of *pmoA* gene (Fig. S2). In general, a much stronger increasing tendency was observed for the methane production of ABSPs, which implied that an increase in MAT more strongly favoured methanogenesis than methanotrophy. Elevated temperature might increase the methane emission from ABSPs through the influence on activities and abundance of functional microbes.

**Figure 6:**
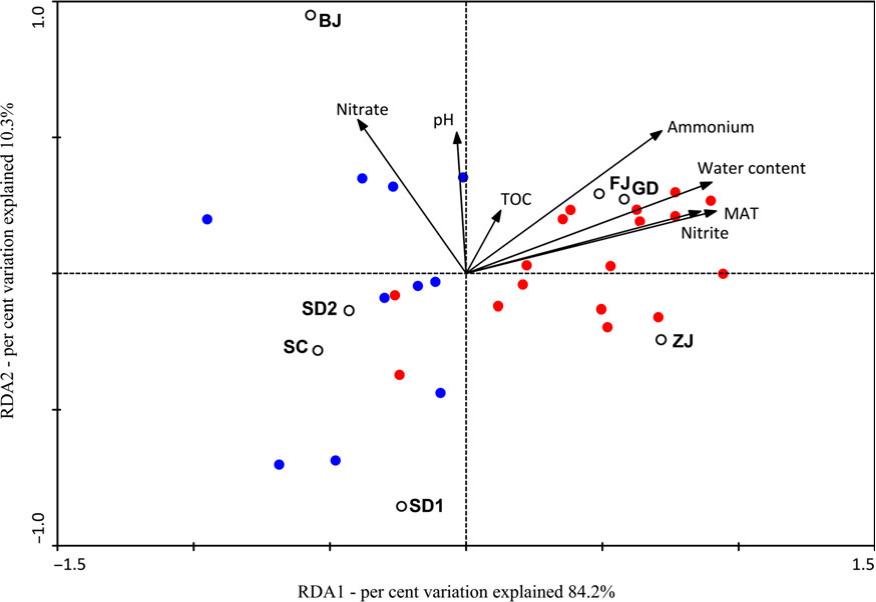
Results of redundancy analysis (RDA) at the family level of archaeal community structure. The blue solid dots represent the dominant families in the sediment samples from LM area. The red ones represent the dominant families in the sediment samples from HM area. The analyses indicated that archaeal community structures of sediment samples collected from LM and HM area were significantly different. The environmental factors in the first two RDA axes explained 84.2% and 10.3% of the total variation of archaeal community structure. Among all the environmental factors, MAT (*P *=* *0.012), water content (*P *=* *0.018) and nitrite concentration (*P *=* *0.028) of sediment samples significantly impacted on the archaeal community structure.

Second, statistical analysis including Pearson's correlation analysis and archaeal redundancy analysis (RDA) demonstrated that MAT played the most important factor in impacting on methanogenesis in ABSPs sediments for the lowest *P* value compared with other factors. Pearson's correlation analysis showed that ammonium of pond water and COD of influent were significantly correlated with the abundance of 16S rRNA gene of methanogens (*P *< 0.05) (Table S5). However, compared with ammonium and COD, MAT presents a stronger significant correlation with the abundance of 16S rRNA gene of methanogens (*P *<* *0.01) (Table S5). Archaeal RDA analyses indicated that archaeal community structures of sediment samples collected from LM and HM area were significantly different (Fig. 6). Among all the environmental factors, the lowest *P* value of MAT (*P *=* *0.012) indicated that MAT was the most important determinant of the archaeal community structure.

**Figure 7:**
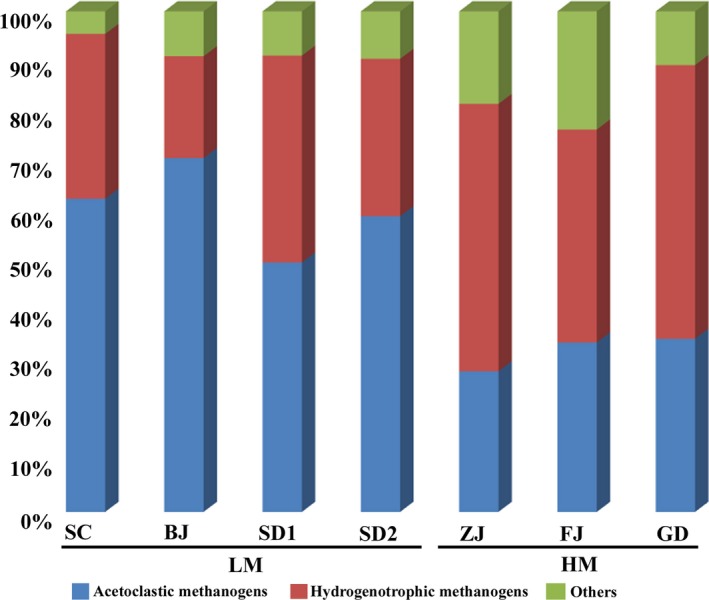
Methanogens distribution in each sediment sample. Different colours represent methanogens with different methanogenic pathways. The red ones are acetoclastic methanogens, the green ones are hydrogenotrophic methanogens, green ones are other types of methanogens and other archaea.

Besides, methanogenic community distribution analyses revealed that methanogenic pathways shifted in the sediment samples from different MAT areas (Fig. 7). Acetoclastic methanogens (49.8–70.7%) were predominant in the sediment samples from LM area while hydrogenotrophic methanogens (42–54.6%) were mainly dominant in the sediment samples from HM area. Strikingly, MAT also influenced the distribution of bacteria in the sediment samples, which could support the distinct methanogenic pathways. For example, *Clostridiaceae*,* Flavobacteriaaceae*,* Turicibacteraceae*,* Anaerolinaceae*,* Actinomycetales* and *Synergistales* (Fernandez *et al*., 2008; Overmann, 2008; Jumas‐Bilak *et al*., 2009; Ito *et al*., 2012; Wang *et al*., 2014), which were well known for their roles in the fermentation of macromolecular organic matter (lipid, cellulose, propionate, glucose, fatty acid and amino acid) into acetate, showed a higher abundance in the bacterial community of the sediment samples from LM area than HM area. *Comamonadaceae* and *Pseudomonadaceae*, which were known to be homo‐acetogens and could provide acetate for acetoclastic methanogens by consuming H_2_ and CO_2_ (Morrill *et al*., 2014), were enriched in the sediment samples from LM area. In contrast, in the sediment samples from HM area, more than 31.9% of bacteria mainly belonged to the phylum of *Bacteroidetes* (Qiu *et al*., 2008) and *Proteobacteria* such as *Syntrophaceae* (Briones *et al*., 2009) and *Syntrophorhabdaceae* (Gray *et al*., 2011), and are known to have a syntrophic relationship with hydrogenotrophic methanogens. *Rikenellaceae*,* Porphyromonadaceae*,* Bacteroidaceae*,* Ruminococcaceae*,* Spirochaetes*,* OP8*,* OD1*,* Peptococcaceae*,* Lentisphaerae* and *Thermotogacaea* (Jehmlich *et al*., 2010; Cheng *et al*., 2013; Zhang *et al*., 2013; Veeravalli *et al*., 2014; Wang *et al*., 2014), which accounted for about 14.2% of the bacteria in the sediments from HM area, were able to produce hydrogen. It has also been reported in reactor experiments that hydrogenotrophic methanogens become more important under higher temperature (55°C) with a syntrophic relationship with acetate‐oxidizing bacteria (Guo *et al*., 2014). Our results further verified this tendency in environmental sediments with a relatively low temperature.

Overall, it could be concluded that MAT is an important factor to the methanogenesis process in ABSPs sediments. With the increasing MAT, methane emission was enhanced mainly due to the shift of methanogenic pathways being constituted by different fractions of methanogens with different methanogenic activities. In addition, the distribution of bacteria in the sediments, which was impacted by MAT, supported our conclusion that the methanogenic pathways shifted from acetoclastic to hydrogenotrophic with increasing MAT.

### Ecological significance

The high methanogenic activity and the dominant role of methanogenesis in the ABSPs sediments in our study suggested that high biodegradable COD concentration of ABSPs could provide sufficient substrate for methanogens and thus might result in large quantities of methane emission from these uncovered ponds. Therefore, considering the important impact of actual loading rates to methanogenesis (Toprak, 1995), it would be better to conduct further research to assess the underlying mechanisms of actual loading rates’ influence on methanogenesis in ABSPs. From the perspective of methane emission control, ABSPs for swine wastewater treatment are not suitable for application in treating anaerobic digestion effluent but more likely to be utilized as the tailwater treatment process.

Moreover, ecological regulation analysis (Röling, 2007) and specific methanogenic activity (SMA) tests (Enright *et al*., 2005) both suggested that a much higher methanogenic activity might be obtained when hydrogenotrophic methanogens were dominant in the environment compared with the acetoclastic methanogens. This is in accordance with our study that the sediment samples from HM area were dominated by hydrogenotrophic methanogenic pathway and showed a higher methanogenic activity than LM area. Therefore, methane emission from ABSPs in HM area will be largely different from LM area. In the future, methane emission from the ABSPs for swine wastewater treatment should be estimated according to the differences of temperature in different areas.

Finally, we confirmed that elevated temperature could increase the methane emission and impact the methanogenesis in ABSPs sediments. According to IPCC, 2013, the global surface temperature showed a warming of 0.85 (0.65–1.06) °C over the period 1880–2012 and was projected to increase by 2.6–4.8°C under ‘Representative Concentration Pathways (RCP) 8.5’ by the end of the 21st century (Pachauri, 2013). Therefore, more methane is expected to be emitted from ABSPs for swine wastewater treatment in response to the increasing temperature, possibly because of a variation of methanogenic pathway in the future.

## Conclusion

To our knowledge, this is the first study to investigate the variation of methanogenesis with a MAT gradient in ABSPs sediments. A high methanogenic activity and abundance of methanogens were observed in the ABSPs sediments. MAT was verified to be the most important factor on methanogenesis. Higher methanogenic activities were obtained in HM compared with LM areas. The acetoclastic methanogenic pathway dominated in LM areas while the hydrogenotrophic pathway prevailed in HM areas. All of these results suggested that methane emission happened in these uncovered ponds and more methane would be emitted in response to the future global warming. Further studies are urgently needed to estimate methane emission from ABSPs all over the world and adjust the application of ABSPs in wastewater treatment to prevent more methane emission.

## Experimental Procedures

### Site description and sampling

In each province, one or two piggeries were chosen. All of these piggeries used a similar wastewater treatment process: Anaerobic digestion followed by ABSPs. At each chosen site, at least three sediment core subsamples were collected using a steel sediment sampler (10 cm in diameter and 200 cm in length). The sediment cores were sliced at 10 cm intervals about 1.5 m beneath the liquid level and mixed to form one composite sample for each site. The composite sediment from each site was then treated as one sediment sample. All the analyses were performed based on these composite samples. Wastewater samples (influent and pond water) were collected using a glass water sampler (1 l). Pond water means the water samples collected at a depth of 0.5 m below the surface (Wilkinson *et al*., 2013). The sediment and water samples were immediately transported on ice to laboratory for analyses. Each sediment sample was split into two parts: one for DNA extraction (stored at −70°C) and another for the analyses of methanogenic activity, methane oxidation activity and physicochemical characteristics (stored at 4°C).

### Physicochemical characteristics

For the analyses of nitrogen compounds, ammonia, nitrite, nitrate and total nitrogen content (TN), the standard methods for the examination of water and wastewater (APHA, 1998) were used. COD was measured by kits (Hach, USA). pH was measured with a pH meter (SG2; Mettler‐Toledo, Greifensee, Switzerland). The sediment ammonium, nitrite and nitrate were extracted from the sediment samples using 2 M KCl with mass–volume ratio of 1:10. Then, water samples were prepared by filtering through a 0.45 μm filter (Kempers and Zweers, 1986) and measured according to the standard methods for the examination of water and wastewater (APHA, 1998). Moisture content was determined by drying 5 g of sediment at 105°C for 10 h. Total organic carbon (TOC) was determined by a TOC analyser (Multi N/C 2100, Jena, Germany). All the physicochemical characteristics were measured in triplicate.

### Methanogenic activity and methane oxidation activity analysis

Methanogenic activity and methane oxidation activity were measured by serum bottle incubation method. The methanogenic activity of sediments was measured in triplicate. About 5 g of fresh sediment samples was placed in 100 ml serum bottles. In order to avoid sampling bias, the sediment was completely mixed before weighing. The serum bottles were flushed with O_2_‐free N_2_ for 3 min and sealed with butyl rubber lids to keep an anaerobic environment. Then the bottles were incubated in static condition at 25°C for 24 h. After incubation, CH_4_ contained in the headspace of the serum bottle was determined by gas chromatography (Shimadzu GC‐14B, FID). The methanogenic activity was calculated and expressed as micromoles of CH_4_ per gram of dry sediment per hour (Dong *et al*., 2013).

Methane oxidation activity was analysed according to the method described by Hanson (1998). Triplicate fresh sediment samples (5 g) were placed in 100 ml serum bottles and then sealed with butyl rubber lids. Each bottle was then injected with 5 ml of highly pure CH_4_ (99.99%) and incubated in static at 25°C for 24 h. CH_4_‐amended bottles without sediments amendment were set as control A. The sediment‐amended bottles without CH_4_ injection were also included (control B) in order to exclude the influence of methane production during the incubation. CH_4_ in the headspace of control and experimental treatments were measured using gas chromatography (Shimadzu GC‐14B, FID). Methane oxidation activity was calculated and expressed as consumed micromoles of CH_4_ per gram of dry sediment per hour (Dong *et al*., 2013). All the physicochemical characteristics were measured in triplicate.

### DNA extraction

In order to avoid DNA extraction bias, the sediment was completely mixed before sampling. Genomic DNA was extracted from 0.5 g of sediment using FastDNA SPIN Kit for Soil (MP Biomedical, LLC, Ohio, USA) according to the manufacturer's protocol. The extracted DNA was stored at −70°C after being examined by 0.8% gel electrophoresis. The concentrations and quality of extracted DNA were determined using a Nanodrop (Thermo Scientific, Wilmington, DE, USA).

### Quantification of functional genes

Quantitative PCR (qPCR) was conducted to estimate the abundances of 16S rRNA gene of methanogens and *pmoA*‐encoding aerobic methane monooxygenase. The quantification was based on the intensity of SYBR Green dye fluorescence. The PCR reaction mixture consisted of 2 μl of template DNA, 0.1 μmol L^−1^ of each primer, 1× SYBR Premix EX Taq (Perfect Real Time) premix reagent (TaKaRa, Dalian, China) and ultrapure DNase/RNase‐free water (ddH_2_O) was added to get a final volume of 20 μl. Primers and thermal cycling used for each reaction were listed in Table S7. PCR reactions were performed in triplicate in a Bio‐Rad CFX1000 Thermal Cycler, with ddH_2_O used as a negative control template. The gene copy numbers were calculated by comparing threshold cycles obtained in each PCR run with standard DNA concentrations (Wang *et al*., 2013). Purified plasmids of 16S rRNA gene of methanogens and *pmoA* gene clones from one sediment sample were used as standard templates to obtain the standard curves.

### Sequencing and data analysis

16S rRNA genes were amplified using the barcoded bacterial primer (520F‐802R) (Milani *et al*., 2013) and barcoded archaeal primer (787F‐1059R) (Lu *et al*., 2012), which targeted the V4 and V5‐V6 region respectively. Illumina sequencing (2 × 150 bp read length) was carried out using the Miseq platform by Personal Biotechnology in Shanghai, China. Illumina paired‐end sequences were filtered and processed using the QIIME package (V 1.7.0) (Caporaso *et al*., 2010). Only reads greater than 150 bp were included in subsequent analyses. Chimera sequences were removed in mothur software (V 1.31.2) using UCHIME method (Edgar *et al*., 2011). OTUs were clustered using the UCLUST implementation in QIIME at the 97% similarity (Edgar, 2010). Taxonomies were assigned using Greengene database (Wang *et al*., 2007) and predominant OTUs were confirmed to be related species by BLAST searcher (http://blast.ncbi.nlm.nih.gov/Blast.cgi). Rarefaction curves and beta diversity were generated by QIIME, while Venn diagram and alpha diversity were generated by Mothur (Schloss *et al*., 2009). Alpha diversity was calculated using observed species, Chao1, Shannon index and Simpson index. Beta diversity was calculated using both unweighted and weighted UNIFRAC (Lozupone and Knight, 2005).

### Statistical analysis

All environmental data, calculated as means and standard deviations, were compared statistically at the level of 5%. Pearson's correlation analysis was applied to assess the association between the quantity of functional genes, microbial community and environmental factors (MAT, NH_4_^+^‐N, NO_2_^−^‐N, NO_3_^−^‐N, TOC, pH, EC and COD), and biological activities (methanogenic activity and methane oxidation activity) using spss 20.0 software. Redundancy analysis (RDA) test was conducted using CANOCO for Windows (version 4.5, Wageningen, the Netherlands) to better understand the potential relationship between distribution of microbial community structure (archaea at the level of family, bacteria at the level of phylum) and environmental factors, including physicochemical characteristics and MAT of sampling sites.

### Accession numbers of nucleotide sequences

The sequencing data discussed in this study were deposited to the SRA databases under accession numbers SPR2127253, SPR2127255, SPR2127260–SPR2127271.

## Conflict of interest

The authors declare no conflict of interest.

## Supporting information


**Fig. S1.** Rarefaction curve of bacterial and archaeal community.
**Fig. S2.** Abundance of 16S rRNA gene of methanogens and *pmoA* gene (copy number L^−1^ water) in the water samples. Error bars indicate standard deviation of the mean of triplicate qPCR reactions.
**Table S1.** Physicochemical characteristics of the water samples.
**Table S2.** Abundance of 16S rRNA gene of methanogens and *pmoA* gene in the sediment samples.
**Table S3.** Detailed distribution of top 3 bacterial phyla in each sediment sample.
**Table S4.** Comparison of Archaeal diversity in all collected sediment samples.
**Table S5.** Correlation between environmental variables and abundance of functional genes involved with methane.
**Table S6.** qPCR primers and thermal cycling conditions.
