# Supplementary material for: Latitudinal distribution of microbial communities in anaerobic biological stabilization ponds: effect of the mean annual temperature
Source: Microb Biotechnol. 2016 Aug 26;9(6):834–45. doi: 10.1111/1751-7915.12407 (PMC5072199; doi:10.1111/1751-7915.12407)
Supplement: Supplementary file 1 — Fig. S1. Rarefaction curve of bacterial and archaeal community. Fig. S2. Abundance of 16S rRNA gene of methanogens and pmoA gene (copy number L−1 water) in the water samples. Error bars indicate standard deviation of the mean of triplicate qPCR reactions. Table S1. Physicochemical characteristics of the water samples. Table S2. Abundance of 16S rRNA gene of methanogens and pmoA gene in the sediment samples. Table S3. Detailed distribution of top 3 bacterial phyla in each sediment sample. Table S4. Comparison of Archaeal diversity in all collected sediment samples. Table S5. Correlation between environmental variables and abundance of functional genes involved with methane. Table S6. qPCR primers and thermal cycling conditions. [file MBT2-9-834-s001.doc]

**Supporting information**

**Tables**

Table S1 Physicochemical characteristics of the water samples.

| Water sample | | | Pond | | | | | | | | | | | Influent | | | | | | | | | | |
| --- | --- | --- | --- | --- | --- | --- | --- | --- | --- | --- | --- | --- | --- | --- | --- | --- | --- | --- | --- | --- | --- | --- | --- | --- |
|  | | Ammonium^a^ | | | Nitrite^a^ | | Nitrate^a^ | | COD^a^ | | DO^a^ | | Ammonium^a^ | | | Nitrite^a^ | | Nitrate^a^ | | COD^a^ | | pH | |  |
| LM | SC | | | 166.67±0.84 | | 0.06±0.00 | | 0.91±0.01 | | 516.67±2.31 | | 0.15±0.01 | | | 168.45±1.84 | | 0.10±0.00 | | 2.05±0.02 | | 663.33±0.57 | | 8.10±0.06 | |
|  | BJ | | | 520.25±2.52 | | 0.08±0.00 | | 1.26±0.02 | | 382.33±4.97 | | 0.17±0.01 | | | 583.10±2.31 | | 0.12±0.00 | | 2.23±0.02 | | 828.00±2.11 | | 8.06±0.07 | |
|  | SD1 | | | 233.93±1.57 | | 0.09±0.00 | | 1.24±0.01 | | 687.00±2.31 | | 0.23±0.01 | | | 382.93±6.87 | | 0.13±0.00 | | 2.41±0.01 | | 1612.00±2.97 | | 8.04±0.09 | |
|  | SD2 | | | 547.03±1.68 | | 0.07±0.00 | | 2.97±0.03 | | 585.33±2.68 | | 0.15±0.01 | | | 608.62±7.01 | | 0.09±0.00 | | 3.58±0.01 | | 1444.70±3.21 | | 8.12±0.10 | |
| HM | ZJ | | | 618.72±13.00 | | 0.13±0.00 | | 0.86±0.01 | | 475.33±1.91 | | 0.15±0.01 | | | 731.56±18.0 | | 0.28±0.01 | | 21.21±0.22 | | 2167.00±2.45 | | 7.12±0.09 | |
|  | FJ | | | 402.98±4.90 | | 0.12±0.00 | | 2.04±0.03 | | 973.33±0.45 | | 0.21±0.02 | | | 500.08±5.10 | | 0.19±0.00 | | 2.67±0.09 | | 2689.30±0.86 | | 7.44±0.08 | |
|  | GD | | | 715.08±12.80 | | 0.10±0.01 | | 0.88±0.12 | | 782.67±0.57 | | 0.17±0.02 | | | 700.42±1.97 | | 0.11±0.01 | | 1.34±0.17 | | 1718±4.09 | | 7.75±0.07 | |

^a^: Means ± standard deviation. The measure unit is mg/l.

Table S2 Abundance of 16S rRNA gene of methanogens and *pmoA* gene in the sediment samples.

| (copies/g dry sediment) | | 16S rRNA gene of methanogens | *pmoA* |
| --- | --- | --- | --- |
| LM | SC | 1.70×10^10^±1.09×10^9^ | 8.59×10^4^±1.44×10^4^ |
|  | BJ | 7.73×10^9^±2.64×10^8^ | 6.95×10^4^±1.20×10^4^ |
|  | SD1 | 1.50×10^10^±2.12×10^9^ | 7.13×10^4^±2.30×10^3^ |
|  | SD2 | 2.43×10^10^±1.60×10^9^ | 1.46×10^5^±3.40×10^4^ |
| HM | ZJ | 5.83×10^10^±5.26×10^9^ | 4.14×10^5^±1.63×10^5^ |
|  | FJ | 8.75×10^10^±1.21×10^10^ | 4.63×10^6^±1.41×10^5^ |
|  | GD | 8.13×10^10^±6.92×10^9^ | 9.78×10^5^±1.79×10^5^ |

Means ± standard deviation.

Table S3 Detailed distribution of top 3 bacterial phyla in each sediment sample.

|  |  |  |  | LM | | | | HM | | |
| --- | --- | --- | --- | --- | --- | --- | --- | --- | --- | --- |
| Phylum | Class | Order | Family | SC | BJ | SD1 | SD2 | ZJ | FJ | GD |
| Firmicutes | Clostridia | Clostridiales | Clostridiaceae | 24.56% | 48.16% | 16.64% | 14.45% | 12.11% | 9.47% | 11.40% |
|  |  |  | Peptostreptococcaceae | 18.80% | 19.12% | 9.43% | 7.13% | 8.38% | 4.51% | 4.23% |
|  |  |  | Unclassified | 0.92% | 1.12% | 1.20% | 1.36% | 1.81% | 1.37% | 1.38% |
|  |  |  | Tissierellaceae | 0.66% | 2.01% | 0.42% | 1.32% | 1.93% | 0.47% | 1.50% |
|  |  |  | Ruminococcaceae | 0.39% | 0.10% | 0.20% | 0.14% | 1.18% | 1.74% | 0.21% |
|  |  |  | Syntrophomonadaceae | 0.40% | 0.28% | 0.31% | 0.35% | 0.74% | 0.27% | 0.65% |
|  |  |  | Lachnospiraceae | 0.44% | 0.49% | 0.50% | 0.52% | 0.44% | 0.54% | 0.07% |
|  |  |  | Christensenellaceae | 0.09% | 0.05% | 0.07% | 0.08% | 0.48% | 0.81% | 0.67% |
|  |  |  | Peptococcaceae | 0.16% | 0.02% | 0.11% | 0.10% | 0.18% | 0.05% | 0.25% |
|  |  |  | Gracilibacteraceae | 0.01% | 0.00% | 0.02% | 0.05% | 0.03% | 0.01% | 0.48% |
|  |  | SHA-98 | Unclassified | 0.03% | 0.03% | 0.04% | 0.04% | 0.86% | 0.81% | 0.29% |
|  |  |  | D2 | 0.06% | 0.00% | 0.07% | 0.00% | 0.03% | 0.06% | 0.26% |
|  | Bacilli | Turicibacterales | Turicibacteraceae | 9.50% | 8.28% | 1.97% | 1.92% | 3.26% | 0.86% | 0.84% |
|  |  | Lactobacillales | Carnobacteriaceae | 0.85% | 0.52% | 0.79% | 0.38% | 0.05% | 0.01% | 0.00% |
|  |  | Bacillales | Planococcaceae | 0.64% | 0.08% | 0.20% | 0.68% | 0.02% | 0.01% | 0.01% |
| Bacteroidetes | Bacteroidia | Bacteroidales | Unclassified | 3.08% | 3.01% | 11.22% | 9.19% | 14.27% | 19.89% | 37.59% |
|  |  |  | SB-1 | 0.18% | 0.01% | 0.71% | 6.72% | 3.77% | 1.63% | 1.38% |
|  |  |  | Rikenellaceae | 0.08% | 0.08% | 0.08% | 0.26% | 2.62% | 0.47% | 0.14% |
|  |  |  | Porphyromonadaceae | 0.12% | 0.05% | 0.31% | 0.32% | 0.69% | 0.58% | 0.54% |
|  |  |  | BA008 | 0.27% | 0.00% | 0.10% | 0.30% | 0.46% | 0.42% | 0.13% |
|  |  |  | Bacteroidaceae | 0.07% | 0.03% | 0.24% | 0.20% | 0.59% | 0.15% | 0.26% |
|  | Flavobacteriia | Flavobacteriales | Flavobacteriaceae | 0.08% | 0.03% | 10.93% | 18.77% | 0.31% | 0.55% | 0.29% |
|  |  |  | Cryomorphaceae | 0.08% | 0.04% | 0.43% | 1.72% | 0.15% | 1.05% | 0.38% |
|  |  |  | Weeksellaceae | 0.05% | 0.00% | 0.15% | 0.21% | 0.00% | 0.01% | 0.01% |
|  | Sphingobacterii | Sphingobacteriales | Sphingobacteriaceae | 0.12% | 0.01% | 0.15% | 0.24% | 0.01% | 0.15% | 0.02% |
|  |  |  | Unclassified | 0.00% | 0.00% | 0.05% | 0.20% | 0.01% | 0.21% | 0.01% |
| Proteobacteria | Deltaproteobacteria | Syntrophobacterales | Syntrophaceae | 3.57% | 0.59% | 1.39% | 2.28% | 7.15% | 7.29% | 6.66% |
|  |  |  | Syntrophorhabdaceae | 0.63% | 0.39% | 0.92% | 0.43% | 0.99% | 0.98% | 1.04% |
|  |  | GW-28 | Unclassified | 0.00% | 0.02% | 0.01% | 0.06% | 0.09% | 0.14% | 0.73% |
|  |  | Desulfobacterales | Desulfobulbaceae | 0.10% | 0.03% | 0.29% | 0.30% | 0.15% | 0.09% | 0.08% |
|  |  | Myxococcales | Unclassified | 0.02% | 0.00% | 0.08% | 0.05% | 0.35% | 0.20% | 0.05% |
|  |  |  | 0319-6G20 | 0.00% | 0.00% | 0.01% | 0.00% | 0.33% | 0.23% | 0.07% |
|  |  | Unclassified | Unclassified | 0.01% | 0.00% | 0.01% | 0.00% | 0.13% | 0.37% | 0.04% |
|  | Betaproteobacteria | Burkholderiales | Comamonadaceae | 3.38% | 0.05% | 4.43% | 5.77% | 0.23% | 0.51% | 0.09% |
|  |  |  | Oxalobacteraceae | 0.58% | 0.00% | 0.13% | 0.12% | 0.01% | 0.01% | 0.00% |
|  |  | Rhodocyclales | Rhodocyclaceae | 0.28% | 0.02% | 0.33% | 0.38% | 0.07% | 0.21% | 0.04% |
|  | Gammaproteobacteria | Pseudomonadales | Pseudomonadaceae | 5.37% | 3.61% | 2.05% | 0.54% | 0.09% | 0.34% | 0.33% |
|  |  |  | Moraxellaceae | 1.31% | 0.25% | 0.29% | 0.57% | 0.14% | 0.07% | 0.01% |
|  |  | Alteromonadales | Unclassified | 0.06% | 0.01% | 0.02% | 0.26% | 0.02% | 2.28% | 0.01% |
|  |  |  | Chromatiaceae | 0.01% | 0.00% | 0.01% | 0.02% | 0.03% | 0.36% | 0.09% |
|  |  | Xanthomonadales | Sinobacteraceae | 0.01% | 0.01% | 0.01% | 0.01% | 0.06% | 0.21% | 0.00% |
|  | Epsilonproteobacteria | Campylobacterales | Helicobacteraceae | 0.10% | 0.10% | 0.24% | 0.13% | 0.88% | 2.35% | 0.93% |
|  |  |  | Campylobacteraceae | 2.51% | 0.05% | 0.05% | 0.04% | 0.23% | 0.13% | 0.02% |
|  | Alphaproteobacteria | Caulobacterales | Caulobacteraceae | 0.03% | 0.03% | 0.15% | 0.51% | 0.01% | 0.07% | 0.01% |

Table S4 Comparison of Archaeal diversity in all collected sediment samples.

| Phylum | Class | Order | Family | Genus | LM | | | | HM | | |
| --- | --- | --- | --- | --- | --- | --- | --- | --- | --- | --- | --- |
|  |  |  |  |  | BJ | SD1 | SD2 | SC | ZJ | FJ | GD |
| Euryarchaeota | Methanobacteria | Methanobacteriales | MSBL1 | *SAGMEG-1* | 0.00% | 0.00% | 0.00% | 0.00% | 0.00% | 0.05% | 0.00% |
|  |  |  | Methanobacteriaceae | *Unclassified* | 0.00% | 0.00% | 0.00% | 0.00% | 0.00% | 0.02% | 0.00% |
|  |  |  |  | *Methanobacterium* | 0.02% | 0.04% | 0.04% | 0.26% | 0.04% | 0.08% | 0.04% |
|  |  |  |  | *Methanobrevibacter* | 0.26% | 0.05% | 0.11% | 0.07% | 0.12% | 0.28% | 0.04% |
|  |  |  |  | *Methanosphaera* | 0.10% | 0.08% | 0.04% | 0.10% | 0.04% | 0.11% | 0.01% |
|  |  |  | WSA2 | *Unclassified* | 0.29% | 0.19% | 0.33% | 0.26% | 1.60% | 4.79% | 3.46% |
|  | Methanococci | Methanococcales | Methanococcaceae | *Methanococcus* | 0.00% | 0.00% | 0.00% | 0.00% | 0.00% | 0.01% | 0.00% |
|  | Methanomicrobia | F99a103 | Unclassified |  | 0.00% | 0.00% | 0.00% | 0.00% | 0.01% | 0.00% | 0.00% |
|  |  | Methanocellales | Unclassified |  | 0.01% | 0.01% | 0.00% | 0.01% | 0.01% | 0.01% | 0.00% |
|  |  |  | Methanocellaceae | *Methanocella* | 0.02% | 0.01% | 0.04% | 0.00% | 0.02% | 0.01% | 0.00% |
|  |  | Methanomicrobiales | Unclassified | *Unclassified* | 12.15% | 28.32% | 21.70% | 26.24% | 11.56% | 7.20% | 6.50% |
|  |  |  | Methanocorpusculaceae | *Methanocorpusculum* | 0.01% | 0.00% | 0.66% | 0.01% | 0.02% | 0.03% | 0.00% |
|  |  |  | Methanomicrobiaceae | *Unclassified* | 0.01% | 0.02% | 0.01% | 0.01% | 0.03% | 0.01% | 0.02% |
|  |  |  |  | *Methanoculleus* | 1.78% | 1.25% | 1.51% | 0.57% | 0.75% | 0.63% | 2.44% |
|  |  |  |  | *Methanogenium* | 0.05% | 0.08% | 0.00% | 0.00% | 0.01% | 0.00% | 0.00% |
|  |  |  |  | *Methanoplanus* | 0.00% | 0.01% | 0.00% | 0.02% | 0.01% | 0.07% | 0.00% |
|  |  |  | Methanoregulaceae | *Unclassified* | 0.07% | 0.12% | 0.29% | 0.07% | 0.10% | 0.09% | 0.03% |
|  |  |  |  | *Candidatus Methanoregula* | 1.26% | 1.86% | 1.86% | 1.53% | 1.39% | 1.59% | 0.59% |
|  |  |  |  | *Methanolinea* | 1.05% | 2.33% | 1.92% | 0.88% | 1.42% | 2.62% | 0.95% |
|  |  |  | Methanospirillaceae | *Unclassified* | 0.04% | 0.00% | 0.04% | 0.02% | 0.06% | 0.03% | 0.01% |
|  |  |  | Methanospirillaceae | *Methanospirillum* | 0.39% | 0.57% | 0.59% | 0.39% | 1.25% | 1.27% | 4.43% |
|  |  | Methanosarcinales | ANME-2D | *Unclassified* | 0.04% | 0.01% | 0.04% | 0.00% | 0.02% | 0.00% | 0.01% |
|  |  |  | ANME-2c | *Unclassified* | 0.00% | 0.00% | 0.03% | 0.00% | 0.00% | 0.00% | 0.00% |
|  |  |  | Methanosaetaceae | *Methanosaeta* | 70.46% | 49.53% | 58.51% | 62.44% | 27.96% | 33.67% | 34.20% |
|  |  |  | Methanosarcinaceae | *Unclassified* | 0.08% | 0.16% | 0.17% | 0.09% | 0.04% | 0.05% | 0.28% |
|  |  |  |  | *Methanomethylovorans* | 0.00% | 0.02% | 0.00% | 0.01% | 0.01% | 0.00% | 0.00% |
|  |  |  |  | *Methanosarcina* | 0.13% | 0.11% | 0.39% | 0.03% | 0.08% | 0.11% | 0.10% |
|  |  | YC-E6 | Unclassified |  | 0.00% | 0.00% | 0.01% | 0.00% | 0.01% | 0.00% | 0.00% |
|  | Thermoplasmata | Thermoplasmatales | Unclassified |  | 0.00% | 0.00% | 0.00% | 0.00% | 0.01% | 0.00% | 0.00% |
|  |  |  | CCA47 | *Unclassified* | 0.00% | 0.00% | 0.00% | 0.00% | 0.00% | 0.00% | 0.00% |
|  |  |  | DHVEG-1 | *Unclassified* | 0.01% | 0.01% | 0.01% | 0.00% | 0.06% | 0.01% | 0.01% |
|  |  |  | Methanomassiliicoccaceae | *Unclassified* | 0.93% | 0.44% | 0.96% | 0.49% | 7.08% | 8.48% | 10.71% |
|  |  |  |  | *Methanomassiliicoccus* | 0.59% | 0.21% | 0.11% | 0.87% | 0.68% | 0.96% | 0.60% |
|  |  |  |  | *vadinCA11* | 0.02% | 0.01% | 0.01% | 0.01% | 0.08% | 0.18% | 0.08% |
| Parvarchaeota | Micrarchaea | Micrarchaeles | Unclassified |  | 0.01% | 0.04% | 0.33% | 0.00% | 0.22% | 0.15% | 0.34% |
|  | Parvarchaea | WCHD3-30 | Unclassified |  | 1.29% | 5.70% | 1.20% | 1.13% | 27.14% | 13.99% | 24.75% |
|  |  | YLA114 | Unclassified |  | 0.03% | 0.04% | 0.03% | 0.01% | 0.02% | 0.20% | 0.04% |
| Crenarchaeota | Aigarchaeota | Unclassified |  |  | 0.00% | 0.00% | 0.00% | 0.00% | 0.02% | 0.00% | 0.02% |
|  | MBGB | Unclassified |  |  | 0.04% | 0.05% | 0.06% | 0.03% | 0.13% | 0.44% | 0.17% |
|  | MCG | Unclassified |  |  | 0.06% | 0.08% | 0.10% | 0.03% | 0.07% | 0.18% | 0.03% |
|  |  | pGrfC26 | Unclassified |  | 3.29% | 4.83% | 6.55% | 2.12% | 3.73% | 5.75% | 3.78% |
|  | Thermoprotei | Unclassified |  |  | 0.00% | 0.04% | 0.00% | 0.00% | 0.02% | 0.07% | 0.03% |
| Thaumarchaeota | Cenarchaeales | Cenarchaeaceae | Nitrosotalea | *Unclassified* | 0.01% | 0.02% | 0.03% | 0.02% | 0.01% | 0.00% | 0.01% |
|  | Nitrososphaerales | Nitrososphaeraceae | Candidatus Nitrososphaera | *Unclassified* | 0.01% | 0.03% | 0.02% | 0.04% | 0.02% | 0.08% | 0.01% |

Table S5 Correlation between environmental variables and abundance of functional genes involved with methane.

|  |  | Pond |  | Influent |  | Sediment |  | MAT | 16S rRNA gene of methanogens |
| --- | --- | --- | --- | --- | --- | --- | --- | --- | --- |
|  |  | Ammonium | COD | Ammonium | COD | Ammonium | TOC |  |  |
| Pond water | COD | .622 |  |  |  |  |  |  |  |
| Influent | Ammonium | .471 | .028 |  |  |  |  |  |  |
|  | COD | .498 | .282 | .607 |  |  |  |  |  |
| Sediment | Ammonium | .833^*^ | .304 | .599 | .341 |  |  |  |  |
|  | TOC | .458 | -.100 | .362 | -.302 | .582 |  |  |  |
| MAT | | .873^*^ | .343 | .485 | .723 | .770^*^ | .226 |  |  |
| 16S rRNA gene of methanogens | | .766^*^ | .334 | .418 | .812^*^ | .630 | .003 | .970^**^ |  |
| *pmoA* | | .473 | .649 | .053 | .746 | .200 | -.554 | .611 | .743 |

Table S6 qPCR primers and thermal cycling conditions.

| Gene target:  Primers | Sequence (5’-3’) | Amplicon size (bp) | Thermal conditions | Reference |
| --- | --- | --- | --- | --- |
| 16S rRNA gene of methanogens:  0357f/0691r | CCCTACGGGGCGCAGCAG | 367 | 50 °C | (Dumont et al., 2014) |
|  | GGATTACARGATTTCAC |  |  |  |
| *pmoA*:  A189f/mb661 | GGNGACTGGGACTTCTGG | 510 | 55 °C | (Watanabe et al., 2004) |
|  | CCGGMGCAACGTCYTTACC |  |  |  |

**Figure legends**

Figure S1. Rarefaction curve of bacterial and archaeal community.

Figure S2. Abundance of 16S rRNA gene of methanogens and *pmoA* gene (copy number L^-1^ water) in the water samples. Error bars indicate standard deviation of the mean of triplicate qPCR reactions.


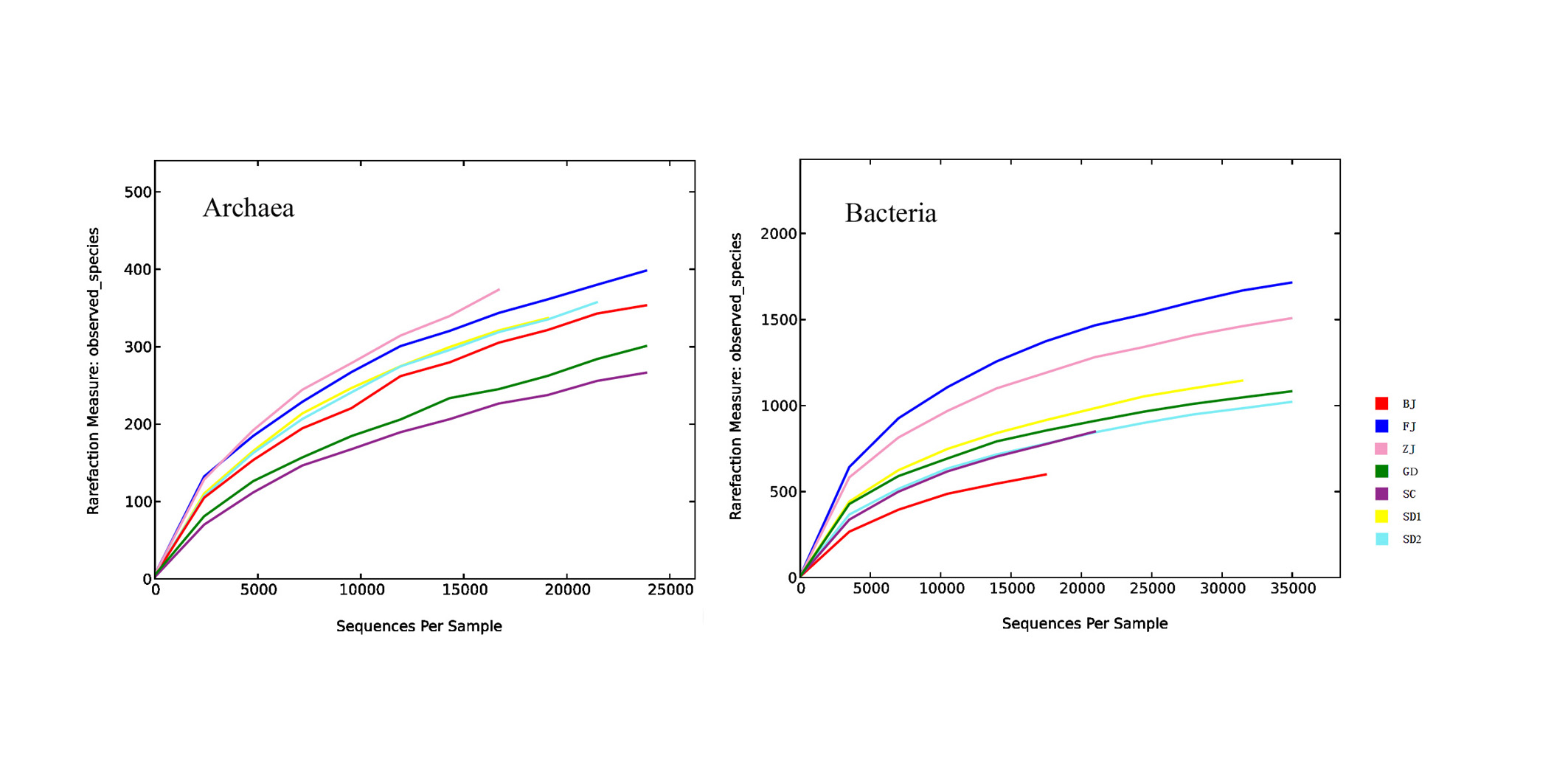


Figure S1.


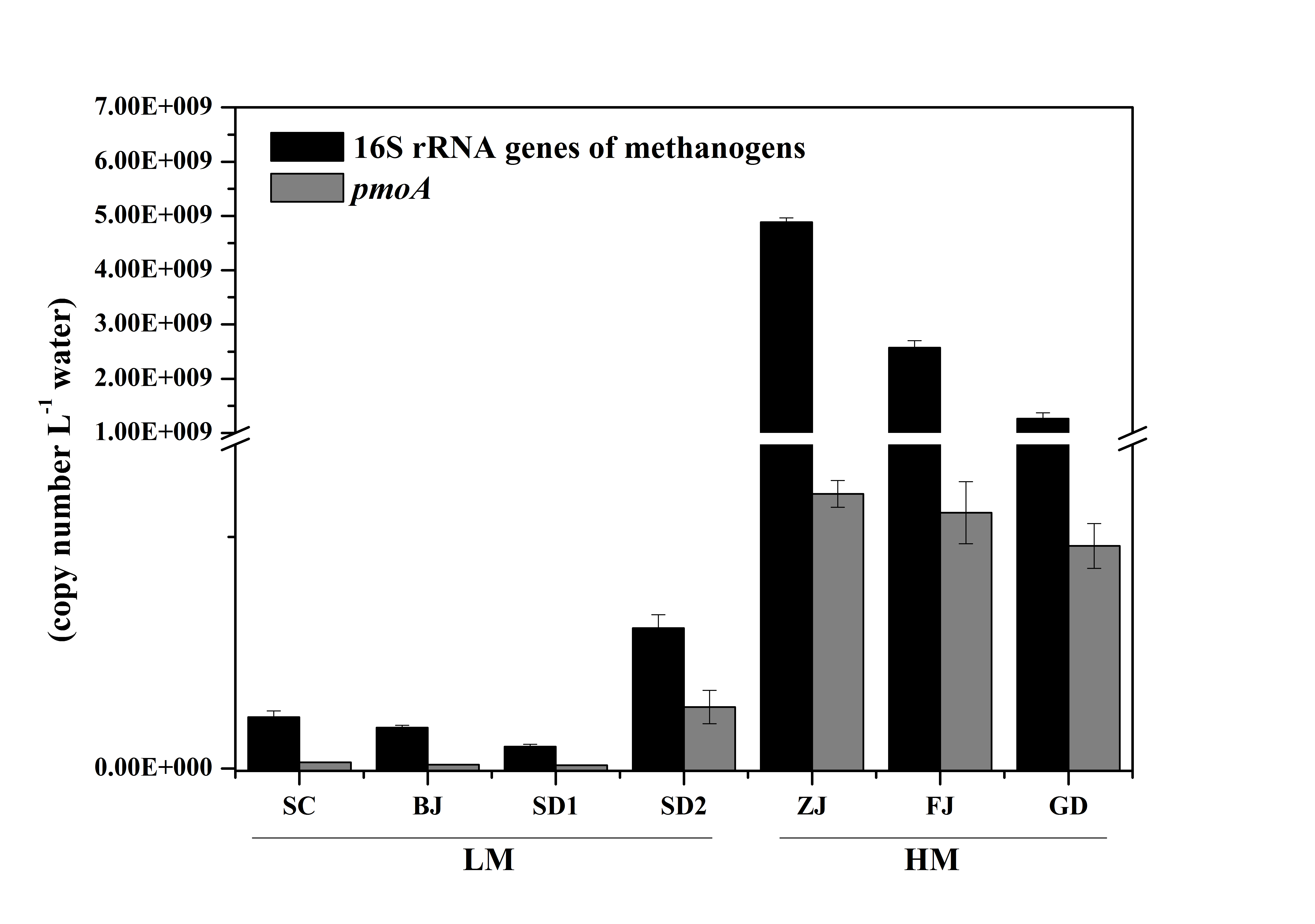


Figure S2.

**References**

Dumont, M.G., McGenity, T., Timmis, K., and Nogales, B. (2014) Primers: functional marker genes for methylotrophs and methanotrophs. *Hydrocarbon and Lipid Microbiology Protocols, eds McGenity TJ, Timmis KN, Nogales B, editors(Berlin: Springer-Verlag*: 1-21.

Watanabe, T., Asakawa, S., Nakamura, A., Nagaoka, K., and Kimura, M. (2004) DGGE method for analyzing 16S rDNA of methanogenic archaeal community in paddy field soil. *FEMS microbiol lett* **232**: 153-163.
